# The anticancer phytochemical rocaglamide inhibits Rho GTPase activity and cancer cell migration

**DOI:** 10.18632/oncotarget.10188

**Published:** 2016-06-20

**Authors:** Michael S. Becker, Paul M. Müller, Jörg Bajorat, Anne Schroeder, Marco Giaisi, Ehsan Amin, Mohammad R. Ahmadian, Oliver Rocks, Rebecca Köhler, Peter H. Krammer, Min Li-Weber

**Affiliations:** ^1^ Tumorimmunology Program (D030), German Cancer Research Center (DKFZ), INF-280, Heidelberg, Germany; ^2^ Max Delbrück Center for Molecular Medicine Berlin-Buch, Berlin, Germany; ^3^ Institute of Biochemistry and Molecular Biology II, Medical Faculty of The Heinrich-Heine University, Düsseldorf, Germany

**Keywords:** flavaglines, rocaglamide, cell migration, Rho GTPase

## Abstract

Chemotherapy is one of the pillars of anti-cancer therapy. Although chemotherapeutics cause regression of the primary tumor, many chemotherapeutics are often shown to induce or accelerate metastasis formation. Moreover, metastatic tumors are largely resistant against chemotherapy. As more than 90% of cancer patients die due to metastases and not due to primary tumor formation, novel drugs are needed to overcome these shortcomings. In this study, we identified the anticancer phytochemical Rocaglamide (Roc-A) to be an inhibitor of cancer cell migration, a crucial event in metastasis formation. We show that Roc-A inhibits cellular migration and invasion independently of its anti-proliferative and cytotoxic effects in different types of human cancer cells. Mechanistically, Roc-A treatment induces F-actin-based morphological changes in membrane protrusions. Further investigation of the molecular mechanisms revealed that Roc-A inhibits the activities of the small GTPases RhoA, Rac1 and Cdc42, the master regulators of cellular migration. Taken together, our results provide evidence that Roc-A may be a lead candidate for a new class of anticancer drugs that inhibit metastasis formation.

## INTRODUCTION

Many chemotherapeutics currently used in anti-cancer treatment mainly act by cytotoxicity. Although, chemotherapy frequently leads to shrinkage in primary tumor volume, several studies have shown that it may also induce or accelerate metastasis formation [1, 2]. One strategy to overcome this shortcoming is to develop small molecule drugs with antimetastatic activity in addition to the cytotoxicity towards cancer cells.

Cancer cell migration is a crucial process in metastasis formation. The first step in cellular migration is polarization of the cell. A leading and trailing edge form in response to an external gradient of signal molecules. In a second step the cell body at the leading edge protrudes and subsequently attaches to the underlying substratum. Eventually, the trailing edge detaches from the substratum and is pulled forward. The migration of the cell and the development of cellular protrusions are largely driven by the reorganization of the actin cytoskeleton [3]. Whereas actin polymerizes at the leading edge of the cell into F-actin, bundled F-actin fibers at the rear of the cell depolymerize. The forming actin meshwork at the leading edge of the cell is the driving force for membrane protrusions, such as the flat and elongated lamellipodia, which play a crucial part in directed cellular migration [4]. Among the main regulators of actin reorganization are the Rho GTPases RhoA, Rac1 and Cdc42 [5, 6]. Rho GTPases shuttle between a GTP-bound active and a GDP-bound inactive form. Loss-of function of any of these molecules has been described to largely inhibit the migratory behavior of cells [7].

The phytochemical Rocaglamide-A (Roc-A) belongs to the chemical class of cyclopenta[b]-tetrahydrobenzofurans, collectively referred to as flavaglines or rocaglamides [8, 9]. *In vivo* and *in vitro* studies have shown that flavaglines/rocaglamides are new candidate drugs for the treatment of cancer [10–14]. So far, the anti-tumor activities of these compounds have been documented to be largely due to inhibition of the eukaryotic translation initiation resulting in blockage of protein translation [12, 15–17]. In addition, a screen involving over 300,000 chemical compounds showed that Roc-A is also a potent inhibitor of HSF1 activation which is involved in cancer glucose uptake [13]. However, whether flavaglines could affect cancer cell migration and metastasis formation has not been thoroughly studied. In this study, we show that Roc-A inhibits cellular migration independent of its anti-proliferative and cytotoxic effects. We show that Roc-A treatment leads to major morphological changes in the organization of F-actin-based protrusions, such as lamellipodia. By applying Förster resonance energy transfer (FRET)-microscopy we revealed that Roc-A reduces the activity of Rho GTPases RhoA, Rac1 and Cdc42. Taken together, our study suggests that Roc-A may be a promising candidate compound for preventing metastasis.

## RESULTS

### Roc-A inhibits cellular migration independent of its cytotoxic and anti-proliferative effects

**Figure 1:**
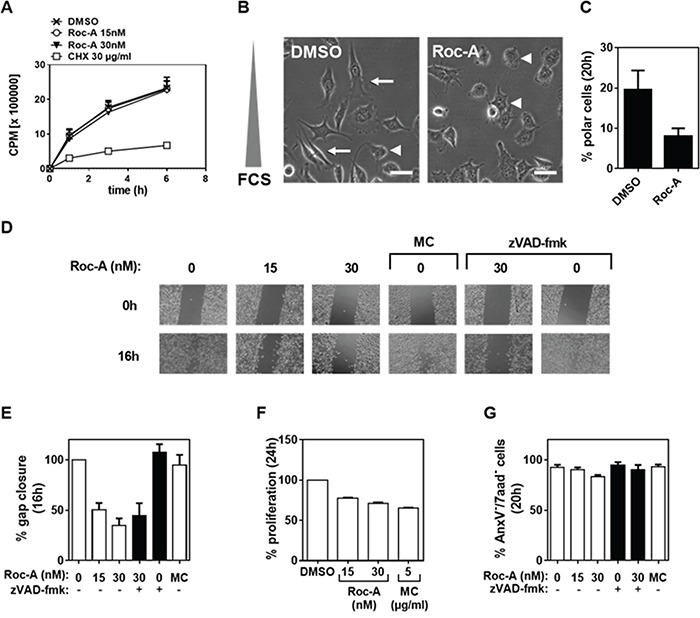
Roc-A inhibits PC-3 cell migration independent of its cytotoxic and anti-proliferative effects **A.** Effect of Roc-A on protein translation. PC3 cells were treated with different doses of Roc-A as indicated. The activities of protein synthesis were monitored by incorporation of ^35^S-methionine. **B.** Roc-A decreases cell polarity in PC-3 cells. PC-3 cells were exposed to a gradient of FCS (0-10%) in the presence of 15 nM Roc-A or solvent (DMSO) for 20 h. Examples of polarized (arrow) and unpolarized (arrowhead) cells are indicated. Scale bare = 50 μm. Representative images are shown. **C.** Quantification of B. At least 230 cells per treatment were analyzed. Results are an average of three independent experiments. Error bars (S.D.) are shown. **D.** Wound assay. A gap was created in confluent PC-3 cell monolayers and then cells were treated with different concentrations of Roc-A in the absence or presence of zVAD (25 μM for 20 min prior to treatment) for 16 h or treated with 5 μg/ml Mitomycin C (MC) for 1h as described in Material and Methods and then further cultured for 16 h. The gaps before (0 h) and after (16 h) treatment are shown. **E.** Quantification of the gap closure. The effects of different drugs on cell migration were quantified as percentage of gap closure. Results are an average of three independent experiments. Error bars (S.D.) are shown. **F.** Analysis of the effects of different drugs on proliferation. PC-3 cells were stained with CFSE as described in Materials and Methods and then seeded and treated as in C. The percentage of proliferative cells was determined and normalized to DMSO treatment. Results are an average of three independent experiments. Error bars (S.D.) are shown. **G.** Analysis of the effects of different drugs on cell viability. The percentage of viable cells was determined as AnxV^−^/7aad^−^ cells 20 h after drug treatment. Results are an average of three independent experiments. Error bars (S.D.) are shown.

We and others have previously shown that Roc-A and its derivatives exert their anticancer effects by inducing apoptosis as well as proliferation arrest (for review see [8]). Along with the study of the anti-proliferative effect of Roc-A [10], we have also observed marked changes in cellular morphology in the prostate cancer cell line PC-3. Under Roc-A treatment, PC-3 cells were less elongated and frequently increased in diameter. To further investigate the influence of Roc-A in cellular morphology, we cultured PC-3 cells in a gradient of FCS ranging from 0 to 10 % in the presence or absence (solvent DMSO) of Roc-A. To exclude the possibility that the observed changes in cellular morphology were due to inhibition of protein synthesis or induction of apoptosis we first examined which doses of Roc-A have no or little effect on translation and cell death. Using an *in vitro* protein synthesis assay, we determined that Roc-A at the concentrations below or equal to 30 nM has no substantial effect on translation inhibition in PC3 cells (Figure 1A). Significant inhibition of protein synthesis by Roc-A was observed at 100 nM and higher (Supplementary Figure S1A). Roc-A also has little effect on apoptosis induction at concentrations below 50 nM (Supplementary Figure S1B). Therefore, we carried out all assays with 15 or 30 nM of Roc-A in PC3 cells.

Consistent with our initial observations, PC3 cells were less elongated when treated with Roc-A (Figure 1B). In addition, Roc-A treatment decreased cell polarity by more than two-fold as determined by the number of cells aligned along the FCS gradient (Figure 1B and 1C). Cell polarity is a crucial and first step in directed cellular migration. Therefore, we hypothesized that Roc-A treatment would prevent cellular migration. To test this hypothesis we first performed a wound-assay. In this assay, confluent cell layers are separated by a gap. Due to the lack of adjacent cells, cells migrate towards each other and close the gap over time. We observed that Roc-A treatment resulted in prevention of gap closure in a dose-dependent manner at concentrations of 15 – 30 nM in PC3 cells (Figure 1D and 1E). As Roc-A can block cellular proliferation by activation of the Chk1/2 signaling pathway [10], we treated PC-3 cells with the anti-proliferative drug Mitomycin C (MC) to exclude the possibility that delayed gap closure was due to inhibition of cellular proliferation. Although MC treatment (5 μg/ml for 1h) led to a stronger inhibition of proliferation than Roc-A treatment (Figure 1F) it did not inhibit gap closure (Figure 1D and 1E). Increased exposure to cells with MC for more than 1h caused a similar level of cell death as Roc-A (Supplementary Figure S1B and S1C). These experiments exclude that the Roc-A-mediated delay in gap closure was due to inhibition of cellular proliferation. In addition to its anti-proliferative effects, Roc-A is also known to be cytotoxic to various cancer cell lines and primary cancer cells by induction of apoptotic cell death. To rule out that the observed delay in gap closure was caused by Roc-A-induced cell death, cell viability was examined by AnxV and 7-AAD staining after treatment. The experiments showed that Roc-A decreased PC3 cell viability by only 5 % when used at 30 nM (Figure 1G). However, gap closure was inhibited by approximately 60% at the same concentration (Figure 1E). In addition, we used the pan-caspase inhibitor zVAD-fmk to block any occurring apoptosis in the assay. In the presence of zVAD-fmk, Roc-A-induced apoptosis was completely blocked (Figure 1G). However, inhibition of apoptosis did not significantly affect the gap closure (Figure 1D and 1E). Taken together, these experiments demonstrate that Roc-A can inhibit cell migration independent from its anti-proliferative and cytotoxic effects.

**Figure 2:**
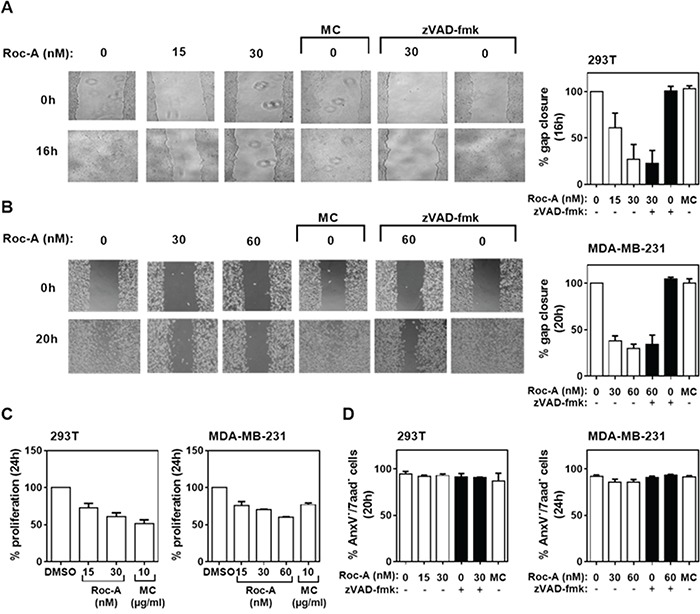
Roc-A inhibits migration of different types of cancer cells **A** and **B.** Roc-A inhibits cell migration in 293T and MDA-MB-231 cells. 293T and MDA-MB-231 cells were subjected to treatment with different drugs and the percentage of gap closure was quantified as described in Figure 1C and 1D. Representative images are shown. **C.** Analysis of the effects of different drugs on proliferation in 293T and MDM-MB-231 cells. The percentage of proliferative cells was determined and normalized to DMSO treatment. Results are an average of three independent experiments. Error bars (S.D.) are shown. **D.** Analysis of the effects of different drugs on cell viability. The percentage of viable cells was determined as AnxV^−^/7aad^−^ cells 20 h (293T) and 24 h (MDA-MB-231) after drug treatment. Results are an average of three independent experiments. Error bars (S.D.) are shown.

To investigate whether Roc-A can inhibit cell migration in general, we tested one normal (non-tumor) fibroblast cell line NIH-3T3 and four additional human cancer cell lines: transformed human embryonic kidney cell line 293T, breast cancer cell line MDA-MB-231, human colon cancer cell line HCT116, and human cervical cancer cell line HeLa. Consistent with the above results, Roc-A blocked gap closures in all cell lines tested in a dose-dependent manner (Figure 2A and 2B; Supplementary Figure S2 and S3). High doses of Roc-A can induce significant apoptotic cell death (Supplementary Figure S4). However, the concentrations of Roc-A used in the wound assays caused only 3-10% cell death (Supplementary Figure S4). In 293T and MDA-MB-231 cells, for instance, Roc-A inhibited gap closure by approximately 70-80% (Figure 2A and 2B). While MC treatment had no effect on gap closure (Figure 2A and 2B), it inhibited cellular proliferation stronger (in 293T cells) or equipotent (in MDA-MB-231 cells) to Roc-A (Figure 2A-2C) and caused higher cell death (10-20%) than Roc-A (Supplementary Figure S5). Cell viability of 293T cells was not affected by Roc-A at concentrations of 15 - 30 nM (Figure 2D; Supplementary Figure S3). In MDA-MB-231 cells, Roc-A treatment resulted in a decrease in cell viability of less than 5%, which could be blocked completely by zVAD-fmk (Figure 2D). Inhibition of apoptosis only marginally affected Roc-A-mediated inhibition of MDA-MB-231 cell migration (Figure 2B). Thus, the anti-migratory effect of Roc-A on PC-3 cells could be reproduced in other cell types suggesting that this effect is cell type-independent.

### Roc-A inhibits cancer cell invasion and impairs directed cellular migration

**Figure 3:**
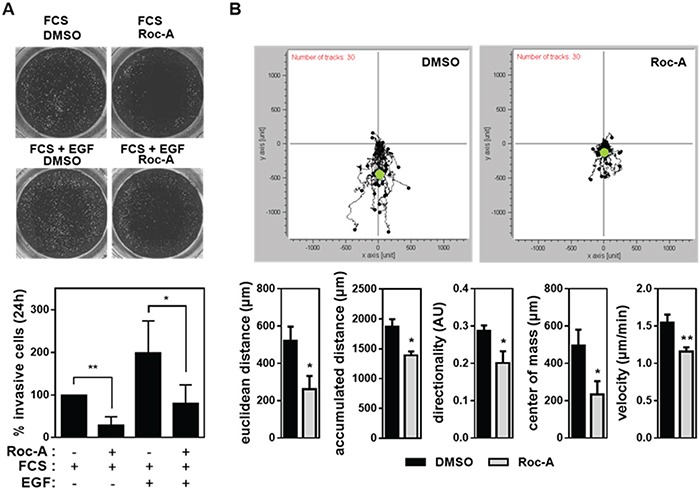
Roc-A inhibits cancer cell invasion and impairs directed cellular migration **A.** Roc-A inhibits PC-3 cell invasion. PC-3 cells were seeded in FCS-free medium on matrigel-coated filters and treated with 15 nM Roc-A or solvent (DMSO). A gradient of FCS and/or EGF was applied by adding 100 ng/ml EGF and/or 5 % FCS to the well below the filter. The upper panel shows the representative image. The lower panel shows the number of invasive cells determined 24 h after treatment and normalized to DMSO-treated cells. Representative images of calcein-stained cells are shown. Results are an average of four independent experiments. Error bars (S.D.) are shown. Asterisks indicate statistical significance with * p < 0.05, **p < 0.01, calculated by unpaired Student's t-test with Welch's correction. **B.** Roc-A impairs directed cellular migration in PC-3 cells. PC-3 cells were exposed to a gradient of FCS (0-10%) and in parallel treated with 15 nM Roc-A or solvent (DMSO). The upper panel shows the movement of 30 cells per treatment tracked over a period of 24 h. Black dots show relative cell positions over 24 h and the green dot indicates the center of mass at the end of the observation period. The lower panel shows the parameters of cellular migration determined. Results are an average of three independent experiments. Error bars (S.D.) are shown. Asterisks indicate statistical significance with * p < 0.05, **p < 0.01, calculated by unpaired Student's t-test with Welch's correction.

In order to form metastasis cancer cells need to leave the primary tumor site via the blood stream or lymphatic system. To reach blood or lymphatic vessels, extracellular matrix (ECM) constituents have to be degraded in a process termed invasion [18]. Therefore, we asked whether Roc-A is able to block the invasive behavior of cancer cells. To investigate this question, we used a Boydon Chamber assay with matrigel as an ECM substituent. PC3 cells were seeded in FCS-free medium on top of matrigel-coated filters in the presence of 15 nM Roc-A or solvent (DMSO). A gradient of FCS and/or EGF was generated by adding 100 ng/ml of EGF and/or 5 % FCS (used as a chemoattractant) to the well below the filter. The experiment showed that treatment of PC-3 cells with Roc-A for 24 h blocked FCS-attracted invasion to more than 70% (Figure 3A). Addition of EGF to FCS led to a twofold increase in cellular invasion, which could also be blocked by Roc-A (Figure 3A). These results indicate that Roc-A can inhibit cellular invasion.

Both, the wound (Figure 1D, 2A and 2B) assay and the invasion (Figure 3A) assay, however, measure cellular migration only indirectly. To directly analyze the effect of Roc-A on cellular migration we performed a live cell imaging experiment. In this experiment PC-3 cells were exposed to a gradient of FCS ranging from 0 to 10% and migration of individual cells were tracked over a time period of 20 h. In average, cells migrated towards the side of the chamber where FCS concentration was highest. In the presence of Roc-A, cellular migration was strongly inhibited (Figure 3B, upper panel). Notably, Roc-A impaired all parameters of cellular migration measured (Figure 3B, lower panel). The strongest impairment was seen for the center of mass and Euclidean distance (Figure 3B, lower panel). The former is a measure for average migration of the entire cell population in direction of FCS gradient and the latter describes the direct distance between the end and starting point of migration of each cell. In conclusion, this experiment indicates that directed migration is inhibited by Roc-A.

### Roc-A alters the morphology of F-actin-based protrusions by an indirect effect on actin polymerization

**Figure 4:**
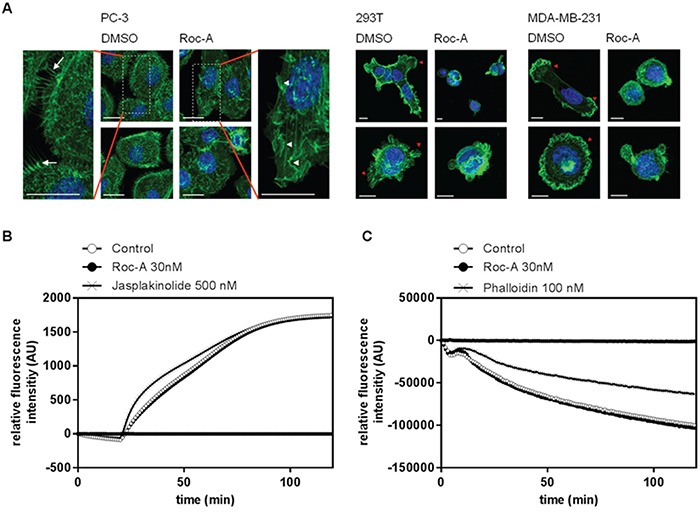
Roc-A alters the morphology of F-actin-based protrusions by an indirect effect on actin polymerization **A.** Roc-A alters the morphology of F-actin-based protrusions. PC-3, MDA-MB-231 and 293T cells were treated with Roc-A (15 nM for PC3, 60 nM for MDA-MB-231 and 30 nM for 293T) or solvent (DMSO) for 24h followed by staining of F-actin (green) and nuclei (blue). Three independent experiments were carried out per cell line. Representative images (Z-stacks) per cell line are shown. Scale bar = 20 μm (PC-3) or 10 μm (293T and MDA-MB-231). **B.** Roc-A does not directly affect actin polymerization. The influence of Roc-A on polymerization of pyrene-conjugated actin monomers was monitored by measuring the increase in fluorescence that occurs upon polymerization of pyrene-conjugated actin. Roc-A, solvent control or the known actin polymerization promoting agent Jasplakinolide were added to pyrene-conjugated actin monomers and 20 min later actin polymerization was initiated. Results are representative of three independent experiments. **C.** Roc-A does not directly affect actin depolymerization. The influence of Roc-A on depolymerization of pyrene-conjugated actin monomers was monitored by measuring the decrease in fluorescence that occurs upon depolymeization of pyrene-conjugated actin. Roc-A, solvent control or the known F-actin stabilizer phalloidin were added to polymerized pyrene-conjugated actin for 20 min, after which depolymerization of actin was initiated. Results are representative of three independent experiments.

One of the most crucial steps in cellular migration is reorganization of the actin cytoskeleton. During migration the monomeric form of actin polymerizes at the leading edge into a branched network of F-actin, while F-actin at the trailing edge is being degraded [19]. Due to the importance of actin reorganization in cellular migration, we further investigated the influence of Roc-A on actin reorganization. To do so, we performed confocal microscopy on F-actin stained cells. PC-3, 293T or MDA-MB-231 cells were treated for 24 h with Roc-A or solvent (DMSO) and cells were subsequently stained for F-actin (Alexa Fluor 488-Phalloidin) and nuclei (DAPI). The experiment showed that Roc-A induced marked changes in F-actin-rich protrusions (Figure 4A). In PC3 cells, while control (DMSO-treated) cells showed filopodia, Roc-A-treated cells largely lacked filopodia and showed increased membrane ruffling (Figure 4A, left panel). In 293T and MDA-MB-231 cells, Roc-A treatment caused a decrease in lamellipodia formation and rounding of cells (Figure 4A, middle and right panel). In summary, Roc-A treatment causes marked changes in cell morphology and F-actin-rich protrusions and the characteristics of these changes are different among different types of cancer cells.

To analyze whether the morphological changes in F-actin-based protrusions were caused by a direct effect of Roc-A on actin polymerization or depolymerization, we performed a cell-free *in vitro* polymerization or depolymerization assay. In the actin polymerization assay, pyrene-conjugated monomeric actin is polymerized, leading to an increase in fluorescence. The actin polymerization-inducer Jasplakinolide was used as a positive control. While Jasplakinolide accelerated the rate of actin polymerization, Roc-A treatment did not differ from the control treatment (Figure 4B). To further analyze whether Roc-A could influence the stability of preformed F-actin filaments directly, we performed a cell-free *in vitro* depolymerization assay. While the known F-actin stabilizing agent Phalloidin strongly delayed F-actin depolymerization, Roc-A did not alter the rate of depolymerizaton (Figure 4C). These results suggest that Roc-A has no direct effect on F-actin stability, neither on actin polymerization nor on actin depolymerization of F-actin filaments.

### Roc-A inhibits the activity of the Rho GTPases RhoA, Rac1 and Cdc42

**Figure 5:**
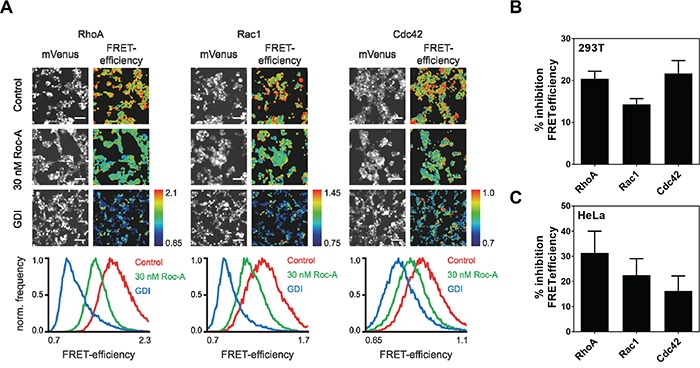
Roc-A inhibits the activity of the Rho GTPases RhoA, Rac1 and Cdc42 **A.** Roc-A inhibits the activity of the Rho GTPases RhoA, Rac1 and Cdc42. RhoA, Rac1 or Cdc42 FRET sensors were overexpressed in 293T cells either with a control plasmid (Control, 30 nM Roc-A) or together with GDI. Cells were treated with 30 nM Roc-A or vehicle (DMSO for Control and GDI) for 24h. Representative fluorescence micrographs show mVenus channel of 293T cells expressing the indicated FRET sensors. Scale bars: 100 μm. Pseudocolored FRET efficiency images of the same field of view were calculated as a ratio of FRET acceptor over FRET donor emission intensity reflecting the GTPase activity levels. The histograms show the pixel distribution of the FRET efficiency within the FRET emission ratio images. **B.** Quantification of A. FRET efficiency was normalized to GDI and DMSO values and % inhibition FRET efficiency was calculated. Results are an average of six independent experiments. Error bars (S.E.M.) are shown. **C.** Roc-A inhibits the activity of the Rho GTPases RhoA, Rac1 and Cdc42 in HeLa cells. RhoA, Rac1 or Cdc42 FRET sensors were overexpressed in HeLa cells either with a control plasmid (Control, 30 nM Roc-A) or together with GDI. Cells were treated with 30 nM Roc-A or vehicle (DMSO for Control and GDI) for 24h. FRET efficiency was normalized to GDI and DMSO values and % inhibition FRET efficiency was calculated. Results are an average of three independent experiments. Error bars (S.E.M.) are shown.

As Roc-A did not influence F-actin polymerization directly (Figure 4B and 4C), we asked whether Roc-A affects activities of the upstream regulators of actin remodeling. The Rho GTPases RhoA, Rac1 and Cdc42 have been shown to be the main upstream regulators of actin remodeling [5, 6]. Therefore, we further investigated the role of Roc-A in regulation of the activities of Rho GTPases by transfection of 293T cells with Förster Resonance Energy Transfer (FRET)-based Rho GTPase sensors for RhoA, Rac1 or Cdc42 [20–22]. The transfected cells were treated with Roc-A or solvent DMSO as control. A decrease in Rho GTPase activity would result in reduction in FRET efficiency which can be monitored by fluorescence microscopy. We also cotransfected FRET-Rho GTPase sensors with excess RhoGDI, a physiologic regulator of Rho GTPase activity that is known to bind and stabilize Rho GTPases in their inactive state [20–23]. We thus created a situation with maximal physiological inhibition of the Rho GTPase activity and comparability between the three Rho GTPases used. As shown in Figure 5A and 5B, Roc-A inhibited the activity of RhoA, Rac1 and Cdc42 by approximately 15 to 22 %, with the strongest effect towards Cdc42 and RhoA. Similar results (approximately 15 to 31% inhibition) were also observed when the experiments were carried out in HeLa cells (Figure 5C). Thus, Roc-A can inhibit the activities of RhoA, Rac1 and Cdc42. To investigate whether the reduced Rho GTPase activity is due to reduction in protein expression, we examined the protein levels in PC3, MDA-MB-231 and 293T cells after Roc-A treatment for 24 h by immunoblot. The analysis showed that Roc-A treatment did not affect the expression levels of the Rho GTPases (Supplementary Figure S6). This is in line with the fact that the concentrations of Roc-A used in this study had little effects on protein translation (Figure 1A and Supplementary Figure S1A).

**Figure 6:**
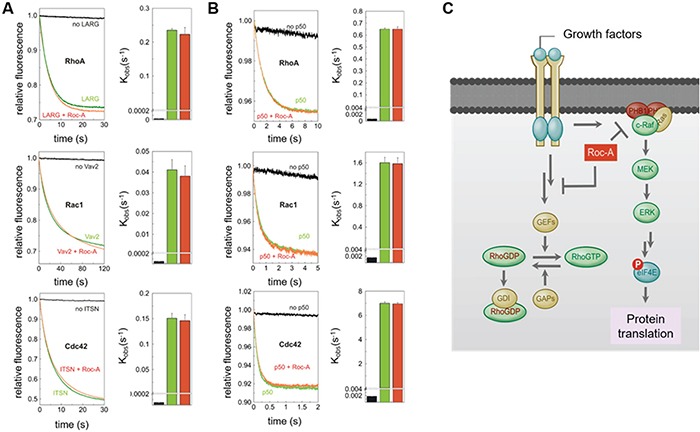
Roc-A does not directly interfere with the activity of GEFs or GAPs **A.** No significant effect of Roc-A on the GEF-accelerated nucleotide exchange of Rho proteins. Dissociation of fluorescent mant-GDP from Rho proteins (RhoA, Rac1 and Cdc42, respectively), accelerated by the RhoGEFs (LARG, Vav2 and ITSN, respectively), was monitored in the absence (green) and in the presence (red) of Roc-A (10 μM). The reactions without RhoGEFs are shown in black. In the right panel, observed rate constants (k_obs_) of the respective measurements are illustrated. **B.** No significant effect of Roc-A on the GAP-accelerated GTP hydrolysis of Rho proteins. GTP hydrolysis of fluorescent tamra-GTP by Rac1 and Cdc42, respectively, and cy3-GTP by RhoA, stimulated by p50GAP, was monitored in the absence (green) and in the presence (red) of Roc-A (10 μM). The reactions in absence of GAP are shown in black. In the right panel, observed rate constants (k_obs_) of the respective measurements are illustrated. All data shown in A and B are an average of four to five different experiments. **C.** Schematic representation of the mechanism by which Roc-A inhibits cellular migration. We hypothesize that Roc-A blocks a signal upstream of Rho-GTPases leading to down-regulation of Rho-GTPase activities and, consequently, inhibition of cellular migration.

Since the activity of Rho GTPases is positively regulated by guanine nucleotide exchange factors (GEFs) and negatively regulated by GTPase activating proteins (GAPs) [24, 25], we asked whether Roc-A could directly interfere with GEF-mediated activation or GAP-mediated inactivation of Rho proteins. To answer this question, we performed an *in vitro* GEF/GAP activity assay based on fluorescent mant-GDP/tamra-GTP bound Rho GTPases [26]. Roc-A was shown to neither affect the activation of Rho GTPases madiated by specific GEFs (LARG, Vav2 and ITSN; Figure 6A) nor the inactivation of Rho GTPases mediated by p50GAP (Figure 6B). These results clearly ruled out the possibility that Roc-A directly interferes with the activity of GEFs or GAPs in general.

Besides GAPs and GEFs, Rho GTPase activity can also be regulated by Rho-specific guanine nucleotide dissociation inhibitor (GDI). GDI binding to Rho GTPases blocks their activity and prevents them from translocating to the plasma membrane [20–22, 27]. To address this possibility, we performed a GDI knockdown experiment. Cells were stably transfected with shRNAs directed against GDI or control. The sequence of shRNA was designed to target an untranslated region of GDI to only interfere with the endogenous GDI expression. The experiment showed that knockdown of GDI did not affect the inhibitory effect of Roc-A on the activity of RhoA (Supplementary Figure S7). Thus, this experiment also ruled out that Roc-A might inhibit Rho GTPase activity through stabilizing the binding of GDI to Rho GTPases.

Whereas RhoA is mainly found in the cytoplasm, Rac1 has been described to be additionally localized at the plasma membrane and Cdc42 only minimally localizes to the plasma membrane [28]. Certain molecules have been described to influence the localization of Rho GTPases [29, 30]. To analyze whether Roc-A may influence the intracellular localization of Cdc42, Rac1 or RhoA, we coexpressed the respective RhoGTPaes together with the plasma membrane marker tH and examined their membrane localization by confocal microscopy. As described in the literature, RhoA was mainly expressed in the cytoplasm, whereas Rac-1 was also localized at the plasma membrane overlapping with tH expression (Supplementary Figure S8). Cdc42 was only partially found at the plasma membrane with clearly dominant cytoplasmic localization. We could not observe any changes in the intracellular localization of all three Rho GTPases upon Roc-A treatment, ruling out that Roc-A interferes with the intracellular localization of Rho GTPases.

In an attempt to find putative upstream pathways with which Roc-A might interfere to attenuate Rho GTPase activity, we looked for known intracellular interaction partners of Roc-A. Recently, we have identified prohibitin (PHB) as the direct target of rocaglamides [16]. Binding of rocaglamides to PHB prevents interaction between PHB and CRaf and, consequently, inhibits CRaf activation and CRaf-MEK-ERK signalling. This action leads to inhibition of protein synthesis [16]. Therefore, we examined the ERK activity at 15 nM Roc-A treatment. Consistent with the translation data (Figure 1A), Roc-A had no influence on ERK activity at the concentration of 15 nM (Supplementary Figure S9A). To further investigate whether the CRaf-MEK-ERK signalling pathway is necessary for Roc-A-mediated inhibition of cell migration, we carried out a siRNA approach to knockdown PHB expression. The experiment showed that knockdown of PHB in PC3 cells had no influence on the effect of Roc-A on inhibition of cell migration (Supplementary Figure S9B and S9C).

In summary, Roc-A inhibits the activation of Rho GTPases Cdc42, Rac1 and RhoA by an indirect effect and independent from the previously described target protein prohibitin.

## DISCUSSION

Metastasis formation is the major cause of death in cancer patients [18]. So far, most cancer treatments are not efficient in fighting metastases because tumors often develop chemo-resistance during therapy [18]. Several studies even show that chemotherapy may enhance metastasis formation [1, 2]. For instance, cisplatin or paclitaxel treatment has been reported to enhance metastasis formation in an *in vivo* lung metastasis model [2]. This side effect highlights a demand for new anticancer drugs that can efficiently kill tumor cells and also inhibit metastasis formation at the same time. In this study, we show that Roc-A may be a promising candidate for such a drug.

To firmly confirm the role of Roc-A in inhibition of cellular migration of cancer cells, we performed three different assays: wound-assay (Figures 1 and 2), invasion assay (Figure 3A) and live cell imaging of cellular migration at very low concentrations (15 to 30 nM) of Roc-A which have no or only minor influence on viability of tumor cells (Figure 3B). All three assays showed a 50 – 80% inhibition of tumor cell migration/invasion after treatment with Roc-A. Roc-A is known to have several other anticancer properties such as inhibition of cell proliferation and induction of apoptosis [10, 15, 16, 31]. Thus, we carried out the assays in the presence of the anti-proliferative drug Mitomycin-C (MC) or the pan-caspase inhibitor zVAD to exclude the possibility that the observed effect of Roc-A on cellular migration was due to its anti-proliferative or cytotoxic activities. We show that Roc-A inhibits tumor cell migration independently of its anti-proliferative or cytotoxic effects by three evidences: First, the anti-proliferative drug MC did not influence cellular migration, although it inhibited proliferation to a similar or even a higher extent than Roc-A (Figures 1D and 2E); second, the concentrations of Roc-A used in cell migration assays caused only 5% or less apoptotic cell death (Figures 1G and 2D); and third, the pan-caspase inhibitor zVAD could not prevent Roc-A-mediated inhibition of gap closure (Figure 1D, 1E and 2A, 2B, 2D). We found that Roc-A can also inhibit migration of non-tumor cells tested on the normal fibroblast NIH-3T3 cells. However, it is not uncommon that anti-metastatic drugs can also block the migration of non-malignant cells. For instance, anti-metastatic Axl-kinase inhibitors are already in clinical trials [32], despite the fact that Axl-deficient primary fibroblasts show reduced migration [33]. In comparison to the more severe side effects observed for most chemotherapeutics (anemia, neutropenia etc.), wound-healing impairment would not be graded as severe. In this regard, we and others have shown that Roc-A is less toxic to many primary non-malignant cells than most chemotherapeutics [8]. Additionally, Roc-A and its derivatives have been tested in several *in vivo* models. Thus far, no adverse toxicities caused by impaired wound healing could be observed. Nevertheless, future *in vivo* studies should particularly monitor for wound healing-related side effects.

Recently, an *in vivo* study with a mouse pancreatic cancer model showed that Roc-A treatment resulted in a significant increase in the lifespan of tumor-bearing mice [34]. In consistence with our study, the study also observed a reduction in tumor metastasis in the lung [34]. However, in that study the concentrations of Roc-A used are quite high and were shown to kill the tumor cells by approximately 50% already. Therefore, it cannot be excluded that the observed effect of Roc-A on metastasis may be largely due to death of cells or inhibition of cell proliferation. In our study, we convincingly demonstrate that Roc-A can inhibit cancer cell migration and invasion independently of its cytotoxic and anti-proliferative activities. Furthermore, our data also show that the inhibitory effect of Roc-A on tumor cell migration is not limited to a specific cancer-type. We have investigated the effect of Roc-A on three different types of cancers: PC-3 (prostate cancer), MDA-MB-231 (breast cancer) and 293T (transformed embryonic kidney). All three cancer types showed a similar response to Roc-A treatment. Thus, Roc-A may be applicable to a wide variety of tumor cells.

In this study, we also further investigated the molecular mechanism by which Roc-A inhibits cellular migration. We found that Roc-A inhibited the activity of all three Rho GTPases (Rac1, RhoA and Cdc42) by approximately 15 to 22% (Figure 5). Rac1, RhoA and Cdc42 are the major regulators of actin remodeling, with their interplay determining the orientation, direction and speed of cellular migration [6, 35]. It has been shown that an increase in only 30% activity of RhoA could cause an increase in cellular migration by approximately 80% [36]. Therefore, we assume that reduction of about 20% of the activities of all three Rho GTPases may lead to a significant reduction of cellular migration. However, we cannot exclude that Roc-A may alter other signaling pathways next to Rho GTPase inhibition that contribute to a reduction in cellular migration. In addition, numerous loss-of-function studies have shown that loss of either of the three Rho GTPases causes a dramatic decrease in cellular migration in many cell types [35]. Coinciding with the inhibition of Rho GTPases by Roc-A, we found that Roc-A treatment induced marked changes in morphology of F-actin-rich protrusions (Figure 4A). Roc-A treatment inhibited formation of filopodia (Figure 4, left panel) and lamellipodia (Figure 4, middle and right panel). Inhibition of Rac1 in MDA-MB-231 cells was shown to result in failure of lamellipodia formation and, subsequently, in cellular migration [37]. Hence, Roc-A-mediated inhibition of Rho GTPases may be one of the molecular mechanisms by which Roc-A causes changes in F-actin-based protrusions (Figure 4) and inhibition of cellular migration (Figures 1–3; Supplementary Figure S2 and S3). Furthermore, reorganization of actin was shown to be necessary for cellular migration [38]. For instance, drugs, such as Cytochalasin D or Jasplinakolide that interfere with actin polymerization directly, strongly inhibit cancer cell migration [39, 40]. Thus, alteration of F-actin morphology by Roc-A is in line with its anti-migratory effect.

In an attempt to unravel the underlying mechanism behind the inhibitory effect of Roc-A on Rho GTPases, we could show that Roc-A did neither influence the expression level nor intracellular localization of Rho GTPases, nor did it interfere with direct binding of GEFs, GAPs or GDIs to Rho GTPases. Consequently, the effect of Roc-A on Rho GTPase activity most likely is being located more upstream in the signaling pathway (Figure 6C). As we could exclude that the inhibitory effect of Roc-A on Rho GTPase activity is mediated by prohibitin or protein translation, another yet undiscovered target of Roc-A must show responsible for the observed inhibitory effect.

So far, the main mechanisms by which Roc-A inhibits cancer cell proliferation have been shown to be due to inhibition of protein synthesis initiation [8] and induction of Cdc25A degradation by activation of the ATM/ATR check point pathway [10]. As shown in our study (Figures 1F and 2C), Roc-A substantially inhibited cell proliferation although the doses used had no or little effect on translation inhibition. Rho GTPases have been described to be involved in regulation of cell cycle progression. Several studies show that inhibition of Rho GTPase activity causes inhibition of cell proliferation [5, 41]. Thus, inhibition of Rho GTPase activity by Roc-A may explain the anti-proliferative effect observed at low concentrations of Roc-A.

Taken together, we show that Roc-A does not only exert cytotoxic and cytostatic effects on cancer cells, but also inhibits tumor cell migration most likely *via* inhibition of activities of Rho GTPases (Figure 6C). Our study strongly suggests that Roc-A may be a lead compound for a new class of chemotherapeutic drugs that kill tumor cells and prevent metastasis at the same time.

## EXPERIMENTAL PROCEDURES

### Cell culture and reagents

The non-tumor fibroblast cell line NIH-3T3, the human cell lines PC-3 (prostate cancer), MDA-MB-231 (breast cancer), the human colon cancer cell line HCT116, the human cervical cancer cell line HeLa, and 293T (transformed embryonic kidney) were cultured at 37°C with 5% CO_2_ in RPMI-1640 medium (Sigma-Aldrich, Munich, Germany) supplemented with 10 % FCS (PC-3 and MDA-MB-231) or in DMEM medium (Sigma-Aldrich) supplemented with 10 % FCS (293T) until otherwise stated in the text. Roc-A (>98 % pure) (Enzo Life Sciences, Lörrach, Germany), Mitomycin-C (Gerbu Biotechnik, Heidelberg, Germany), zVAD-fmk (Bachem, Bubendorf, Switzerland), recombinant human EGF (BioVision, Milpitas, USA), Jasplakinolide (VWR international, Darmstadt, Germany) and Phalloidin (Biotrend, Köln, Germany) were used as indicated in the respective figure legends.

### Wound assay

Cells were seeded in wound assay cell culture inserts (Ibidi, Munich, Germany) and cultivated until 100 % confluence was reached. Subsequently, inserts were removed and cell layers were washed with phosphate buffered saline (PBS), followed by addition of fresh medium containing either Mitomycin-C, solvent (DMSO) as control or Roc-A. After 1 h Mitomycin-C was washed out and fresh medium was added. ZVAD-fmk was added to indicated samples 20 min prior to adding solvent (DMSO) or Roc-A. Pictures of the gap between the two cell layers were taken directly after addition of drugs and at the end of the indicated incubation time. The change in cell-free surface over time was quantified by use of TScratch [42] and normalized to DMSO-treated control samples.

### Determination of cell viability

Cells that were previously analyzed in wound assays, were detached by trypsinization and washed once with Annexin-V binding buffer (0.01 M Hepes, 2.5 mM CaCl_2_, 0.14 M NaCl). Subsequently, cells were stained with 7-amino-actinomycin D (7-aad; Sigma-Aldrich) and Annexin-V–FITC antibody (Immunotools, Friesoythe, Germany) at 4°C for 30 min, followed by a wash step and quantification of Annexin-V/7-aad double-negative cells by flow cytometry.

### Determination of cell proliferation

Cells were labeled with carboxyfluorescein succinimidyl ester (CFSE; Molecular Probes/Thermo Fisher Scientific, Waltham, USA) by incubation of 2 × 10^6^ cells with CFSE (2.5 μM) for 10 min at 37°C in the dark. Incorporation was stopped by addition of ice-cold FCS (10% in PBS). Subsequently, cells were washed three times and were employed in wound assays. 24 h after drug treatment, the amount of incorporated CFSE was quantified by flow cytometry. Proliferation data was normalized to DMSO-treated samples.

### Invasion assay

Invasion assays were carried out according to the manufacturer's instructions. Briefly, 8.5 × 10^4^ cells were plated on top of each rehydrated culture insert (BD BioCoat™ Matrigel™ Invasion Chamber; BD Biosciences, Heidelberg, Germany) in FCS-free medium. Following an incubation time of 24 h, remaining cells in the upper compartment were removed and cells that had migrated through Matrigel™ were stained with Calcein (4 μM; Sigma-Aldrich), followed by quantification as previously described [43].

### Chemotaxis assay

For 2D chemotaxis assays “μ-Slide Chemotaxis”-chambers (Ibidi, Munich, Germany) were used. Chemotaxis assays were carried out according to the manufacturer's instructions. Cells that divided or died during the observation period were excluded from analysis. Images were taken every 5 min for an observation period of 20 h. ImageJ [44] was used for tracking of cells and Chemotaxis and Migration Tool (Ibidi, Munich, Germany) was used for evaluating tracked cell data.

### Confocal microscopy

0.4 × 10^4^ – 0.4 × 10^5^ cells were seeded in each well of a 8-well chamber glass slide (VWR International, Darmstadt, Germany), followed by starvation of cells for 24h in FCS-free medium. In parallel, cells were treated as indicated in the figure legends after which 10 % FCS (in medium) was added for 30 min to stimulate growth factor signaling. Subsequently, cells were washed with PBS twice, fixed with formaldehyde (3%, 15 min), washed three times again with PBS and permeabilized with Triton-X 100 (0.2 %, 10 min). Three PBS wash steps were followed by staining actin with acti-stain 488 phalloidin (Tebu, Offenbach, Germany) for 30 min at room temperature. DAPI mounting medium (Dianova, Hamburg, Germany) was used to stain nuclei.

### Actin (De)polymerization assay

Actin (De)Polymerization Assays were carried out according to manufacturer's instructions using Actin Polymerization Biochem Kit (Cytoskeleton Inc, Denver, USA). Fluorescence was measured for a total time of 120 min with 60 sec intervals. Baseline fluorescence values were established by measurement of unpolymerized pyrene actin or polymerized pyrene actin, respectively, for 5 min, after which drugs or solvent were added. For actin polymerization experiments, actin polymerization was initiated by addition of 10 x Actin Polymerization Buffer (500 mM KCl, 20 mM MgCl2, 10 mM ATP) 20 min after drugs were added.

### Rho GTPase activity measurement – FRET imaging

To study the effect of Roc-A on Rho GTPase activity, 293T cells were transiently transfected with RhoA, Rac1 and Cdc42 FRET sensors [20–22, 45] for 40 h. Each Rho GTPase used in these measurements is labeled with a donor and an acceptor fluorophore. Upon activation of the Rho GTPase, the donor and acceptor fluorophores get into close proximity causing an increase in FRET efficiency. Imaging was performed on an inverted microscope (IX81, Olympus) equipped with a xenon arc burner epifluorescence illumination system (MT20, Olympus), a fluorescence emission filter wheel (Olympus) and an EM-CCD camera (ImagEM, Hamamatsu). Images were taken with a 10x/NA 0.4 Super Apochromat air objective (Olympus) enabling the simultaneous analysis of multiple cells. FRET images were taken with the following settings (excitation, dichroic mirror, emission): FRET-donor 430/25, zt442RDC, 483/32; FRET-acceptor 430/25, zt442RDC, 542/27. Images were analyzed using Image J software. Images were background corrected and regions of interest were defined by FRET-sensor Venus intensity. Average intensities of FRET-acceptor channel were divided by FRET-donor channel to calculate FRET efficiency. To study the effect of Roc-A on Rho GTPase activity in HeLa cells, 1.25 × 10^4^ cells were seeded into each well of a 96 well plate (μ-plate, ibidi). The next day, cells were transiently transfected with FRET sensors for RhoA, Rac1 and Cdc42, together with a control plasmid or GDI for 24h. Cells were then treated with 30 nM Roc-A or vehicle (DMSO for Control and GDI) for 24h. Images were taken using a 20x/NA 0.75 UV Apochromat objective (Olympus). FRET ratio was normalized to the DMSO control.

### Translation assay

The rate of protein synthesis was determined by measuring the amount of incorporated ^35^S-methionine. Briefly, cells were incubated for 3 h in methionine-free medium, followed by addition of 7 μCi of ^35^S-methionine-labeling mix (PerkinElmer, Waltham, MA, USA) per well. An incubation of indicated times was followed by washing with PBS and lysis in ice-cold lysis buffer for 15 min on ice. After clearing of cell lysates 50 μl of each lysate was incubated in 1 ml of Liquid Scintillation Cocktail solution (Beckman coulter, Brea, CA, USA) and radioactivity was determined by Liquid Scintillation counting.

### siRNA knockdown experiment

PC-3 cells were transiently transfected with siRNA directed against PHB (FlexiTube siRNA Hs_PHB_6 [Qiagen, Hilden, Germany]) by use of Lipofectamine 2000 according to the manufacturer's instructions. Transfections were performed in Opti-MEM-GlutaMAX Medium (Thermo Fisher, Waltham, MA, USA).

### GEF and GAP activity measurements

Human RhoA (aa 1-181), Rac1 (aa 1-184), Cdc42 (aa 1-178), the catalytic DH-PH tandem domain of Vav2 (aa 168–543), ITSN (aa 1229–1580), LARG (aa 766–1138) and GAP domain of p50 (aa 198-439) were produced as glutathione S-transferase (GST) fusion proteins in *Escherichia coli* as described previously [26]. Rho protein preparation, including nucleotide-free and fluorescent methylanthraniloyl (mant) GDP/GTP-bound Rho proteins, were prepared as described before [46]. For kinetic measurement, Fluorescence measurements were performed at 25°C in a buffer, containing 30 mM Tris/HCl, pH 7.5, 10 mM K_2_HPO_4_/KH_2_PO_4_, pH 7.5, 5 mM MgCl_2_, and 3 mM dithiothreitol. Changes in fluorescent intensity were monitored in real-time using a stopped-flow instrument (HiTech Scientific SF-61) with a mercury xenon light source and TgK Scientific Kinetic Studio software. For measurement of GEF activity, the dissociation of mant-GDP from Rho proteins (0.2 μM) in the absence and presence of the DH-PH domain (2 μM) of the respective GEFs and excess amounts of GDP (20 μM) was monitored (in absence and presence of Roc-A) [47]. An excitation wavelength of 366 nm was used and emission was detected using a cutoff filter of 408 nm. For measurement of GAP activity, fluorescent GTP-bound Rho proteins (pre-mixing 0.3 μM nucleotide-free Rho and 0.2 μM tamra-/cy3-GTP) and the catalytic domain of p50GAP (2 μM) were rapidly mixed by stopped-flow spectrophotometer (in absence and presence of Roc-A) [48]. Excitation wavelengths of 546 nm and 550 nm were used for tamra and cy3 fluorophores, respectively, and a 570 nm (tamra and cy3) cut-off-filter (Schott glass) was used to collect emitted light. The observed rate constants were calculated by fitting the data as single exponential decay using the GraFit program (Erithacus software).

### Localization of Rho GTPases after RocA treatment

1.25 × 10^4^ cells (HeLa) were seeded on each well of an 8 well slide (μ-slide, ibidi). The next day, cells were transiently transfected with mCitrine-RhoA, mCitrine-Rac1 or mCitrine-Cdc42 each together with mCerulean-tH which comprises only the C-terminal 10 amino acids of HRas and, thus, served as a plasma membrane marker [49, 50]. Cells were treated with 30 nM Roc-A or vehicle (DMSO for Control and GDI) for 24h. Images were taken using a SP8 inverted confocal microscope equipped with a HC PL APO 63x/NA 1.3 glycerol objective. Representative images (Z-slices) are shown.

### GDI-knockdown by shRNA lentiviral vector

To generate cell lines with stable RNAi knockdown of GDI, lentiviral shRNA-mediated stable gene silencing cells was done in 293T as described [51]. CGTCTAACCATGATGCCTTAA was used as targeting sequence in the shRNA and the 1.9 kb stuffer sequence as control. 5.5 × 10^4^ cells of each cell line were seeded on each well of a 96 well plate (μ-plate, ibidi). The next day, cells were transiently transfected with FRET sensors for RhoA, Rac1 and Cdc42, together with a control plasmid or GDI for 24h. Cells were then treated with 30 nM Roc-A or vehicle (DMSO for Control and GDI) for 24h. FRET ratio was normalized to DMSO control. Results are an average of four experiments.

## SUPPLEMENTARY MATERIALS



## References

[1] Park SI, Liao J, Berry JE, Li X, Koh AJ, Michalski ME, Eber MR, Soki FN, Sadler D, Sud S, Tisdelle S, Daignault SD, Nemeth JA (2012). Cyclophosphamide creates a receptive microenvironment for prostate cancer skeletal metastasis. Cancer Res.

[2] Daenen LGM, Roodhart JML, van Amersfoort M, Dehnad M, Roessingh W, Ulfman LH, Derksen PWB, Voest EE (2011). Chemotherapy enhances metastasis formation via VEGFR-1-expressing endothelial cells. Cancer Res.

[3] Gardel ML, Schneider IC, Aratyn-Schaus Y, Waterman CM (2010). Mechanical integration of actin and adhesion dynamics in cell migration. Annu. Rev. Cell Dev. Biol.

[4] Yamaguchi H, Condeelis J (2007). Regulation of the actin cytoskeleton in cancer cell migration and invasion. Biochim. Biophys. Acta - Mol. Cell Res.

[5] Vega FM, Ridley AJ (2008). Rho GTPases in cancer cell biology. FEBS Lett.

[6] Ridley AJ (2001). Rho GTPases and cell migration. J. Cell Sci.

[7] Ridley A (2015). Rho GTPase signalling in cell migration. Curr. Opin. Cell Biol.

[8] Li-Weber M (2015). Molecular mechanisms and anti-cancer aspects of the medicinal phytochemicals rocaglamides (=flavaglines). Int. J. Cancer.

[9] Ebada SS, Lajkiewicz N, Porco JA, Li-Weber M, Proksch P (2011). Chemistry and biology of rocaglamides (= flavaglines) and related derivatives from aglaia species (meliaceae). Prog. Chem. Org. Nat. Prod.

[10] Neumann J, Boerries M, Köhler R, Giaisi M, Krammer PH, Busch H, Li-Weber M (2014). The natural anticancer compound rocaglamide selectively inhibits the G1-S-phase transition in cancer cells through the ATM/ATR-mediated Chk1/2 cell cycle checkpoints. Int. J. Cancer.

[11] Becker MS, Schmezer P, Breuer R, Haas SF, Essers MA, Krammer PH, Li-Weber M (2014). The traditional Chinese medical compound Rocaglamide protects nonmalignant primary cells from DNA damage-induced toxicity by inhibition of p53 expression. Cell Death Dis.

[12] Cencic R, Carrier M, Galicia-Vázquez G, Bordeleau M-E, Sukarieh R, Bourdeau A, Brem B, Teodoro JG, Greger H, Tremblay ML, Porco JA, Pelletier J (2009). Antitumor activity and mechanism of action of the cyclopenta[b]benzofuran, silvestrol. PLoS One.

[13] Santagata S, Mendillo ML, Tang Y, Subramanian A, Perley CC, Roche SP, Wong B, Narayan R, Kwon H, Koeva M, Amon A, Golub TR, Porco JA (2013). Tight coordination of protein translation and HSF1 activation supports the anabolic malignant state. Science.

[14] Boussemart L, Malka-Mahieu H, Girault I, Allard D, Hemmingsson O, Tomasic G, Thomas M, Basmadjian C, Ribeiro N, Thuaud F, Mateus C, Routier E, Kamsu-Kom N (2014). eIF4F is a nexus of resistance to anti-BRAF and anti-MEK cancer therapies. Nature.

[15] Sadlish H, Galicia-Vazquez G, Paris CG, Aust T, Bhullar B, Chang L, Helliwell SB, Hoepfner D, Knapp B, Riedl R, Roggo S, Schuierer S, Studer C (2013). Evidence for a Functionally Relevant Rocaglamide Binding Site on the eIF4A-RNA Complex. ACS Chem. Biol.

[16] Polier G, Neumann J, Thuaud F, Ribeiro N, Gelhaus C, Schmidt H, Giaisi M, Köhler R, Müller WW, Proksch P, Leippe M, Janssen O, Désaubry L (2012). The natural anticancer compounds rocaglamides inhibit the Raf-MEK-ERK pathway by targeting prohibitin 1 and 2. Chem. Biol.

[17] Bordeleau M, Robert F, Gerard B, Lindqvist L, Chen SMH, Wendel H, Brem B, Greger H, Lowe SW, Pelletier J (2008). Therapeutic suppression of translation initiation modulates chemosensitivity in a mouse lymphoma model. Cancer.

[18] Valastyan S, Weinberg RA (2011). Review Tumor Metastasis: Molecular Insights and Evolving Paradigms. Cell.

[19] Wehrle-Haller B, Imhof BA (2003). Actin, microtubules and focal adhesion dynamics during cell migration. Int. J. Biochem. Cell Biol.

[20] Fritz RD, Letzelter M, Reimann A, Martin K, Fusco L, Ritsma L, Ponsioen B, Fluri E, Schulte-Merker S, van Rheenen J, Pertz O (2013). A Versatile Toolkit to Produce Sensitive FRET Biosensors to Visualize Signaling in Time and Space. Sci. Signal.

[21] Hanna S, Miskolci V, Cox D, Hodgson L (2014). A New Genetically Encoded Single-Chain Biosensor for Cdc42 Based on FRET, Useful for Live-Cell Imaging. PLoS One.

[22] Moshfegh Y, Bravo-Cordero JJ, Miskolci V, Condeelis J, Hodgson L (2014). A Trio-Rac1-Pak1 signalling axis drives invadopodia disassembly. Nat. Cell Biol.

[23] Pertz O, Hodgson L, Klemke R, Hahn K (2006). Spatiotemporal dynamics of RhoA activity in migrating cells. Nature.

[24] Vigil D, Cherfils J, Rossman KL, Der CJ (2010). Ras superfamily GEFs and GAPs: validated and tractable targets for cancer therapy?. Nat. Rev. Cancer.

[25] Dvorsky R, Ahmadian MR (2004). Always look on the bright site of Rho: structural implications for a conserved intermolecular interface. EMBO Rep.

[26] Eberth A, Ahmadian MR (2009). In vitro GEF and GAP assays. Curr. Protoc. Cell Biol.

[27] Pertz O (2010). Spatio-temporal Rho GTPase signaling - where are we now?. J. Cell Sci.

[28] Michaelson D, Silletti J, Murphy G, D'Eustachio P, Rush M, Philips MR (2001). Differential localization of Rho GTPases in live cells: regulation by hypervariable regions and RhoGDI binding. J. Cell Biol.

[29] Fibbi B, Morelli A, Marini M, Zhang X-H, Mancina R, Vignozzi L, Filippi S, Chavalmane A, Silvestrini E, Colli E, Adorini L, Vannelli GB, Maggi M Atorvastatin but not elocalcitol increases sildenafil responsiveness in spontaneously hypertensive rats by regulating the RhoA/ROCK pathway. J. Androl.

[30] Ruiz-Velasco R, Lanning CC, Williams CL (2002). The activation of Rac1 by M3 muscarinic acetylcholine receptors involves the translocation of Rac1 and IQGAP1 to cell junctions and changes in the composition of protein complexes containing Rac1, IQGAP1, and actin. J. Biol. Chem.

[31] Li-Weber M (2013). Targeting apoptosis pathways in cancer by Chinese medicine. Cancer Lett.

[32] Myers SH, Brunton VG, Unciti-Broceta A (2016). AXL Inhibitors in Cancer: A Medicinal Chemistry Perspective. J. Med. Chem.

[33] Linger RMA, Keating AK, Earp HS, Graham DK (2008). TAM receptor tyrosine kinases: biologic functions, signaling, and potential therapeutic targeting in human cancer. Adv. Cancer Res.

[34] Luan Z, He Y, Alattar M, Chen Z, He F (2014). Targeting the prohibitin scaffold-CRAF kinase interaction in RAS-ERK-driven pancreatic ductal adenocarcinoma. Mol. Cancer.

[35] Heasman SJ, Ridley AJ (2008). Mammalian Rho GTPases: new insights into their functions from in vivo studies. Nat. Rev. Mol. Cell Biol.

[36] Seo M, Lee W, Suk K (2010). Identification of novel cell migration-promoting genes by a functional genetic screen. FASEB J.

[37] Montalvo-Ortiz BL, Castillo-Pichardo L, Hernández E, Humphries-Bickley T, De la Mota-Peynado A, Cubano LA, Vlaar CP, Dharmawardhane S (2012). Characterization of EHop-016, novel small molecule inhibitor of Rac GTPase. J. Biol. Chem.

[38] Insall RH, Machesky LM (2009). Actin dynamics at the leading edge: from simple machinery to complex networks. Dev. Cell.

[39] Fenteany G, Zhu S (2003). Small-molecule inhibitors of actin dynamics and cell motility. Curr. Top. Med. Chem.

[40] Yarrow JC, Totsukawa G, Charras GT, Mitchison TJ (2005). Screening for cell migration inhibitors via automated microscopy reveals a Rho-kinase inhibitor. Chem. Biol.

[41] Yoshida T, Zhang Y, Rivera Rosado LA, Chen J, Khan T, Moon SY, Zhang B (2010). Blockade of Rac1 activity induces G1 cell cycle arrest or apoptosis in breast cancer cells through downregulation of cyclin D1, survivin, and X-linked inhibitor of apoptosis protein. Mol. Cancer Ther.

[42] Gebäck T, Schulz M.M.P, Koumoutsakos P, Detmar M (2009). A novel and simple software tool for automated analysis of monolayer wound healing assays. Biotechniques.

[43] Brockschmidt A, Trost D, Peterziel H, Zimmermann K, Ehrler M, Grassmann H, Pfenning PN, Waha A, Wohlleber D, Brockschmidt FF, Jugold M, Hoischen A, Kalla C (2012). KIAA1797/FOCAD encodes a novel focal adhesion protein with tumour suppressor function in gliomas. Brain.

[44] Schneider CA, Rasband WS, Eliceiri KW (2012). NIH Image to ImageJ: 25 years of image analysis. Nat. Methods.

[45] Zadran S, Standley S, Wong K, Otiniano E, Amighi A, Baudry M (2012). Fluorescence resonance energy transfer (FRET)-based biosensors: visualizing cellular dynamics and bioenergetics. Appl. Microbiol. Biotechnol.

[46] Jaiswal M, Dubey BN, Koessmeier KT, Gremer L, Ahmadian MR (2012). Biochemical assays to characterize Rho GTPases. Methods Mol. Biol.

[47] Jaiswal M, Gremer L, Dvorsky R, Haeusler LC, Cirstea IC, Uhlenbrock K, Ahmadian MR (2011). Mechanistic insights into specificity, activity, and regulatory elements of the regulator of G-protein signaling (RGS)-containing Rho-specific guanine nucleotide exchange factors (GEFs) p115, PDZ-RhoGEF (PRG), and leukemia-associated RhoGEF (LARG). J. Biol. Chem.

[48] Jaiswal M, Dvorsky R, Amin E, Risse SL, Fansa EK, Zhang S-C, Taha MS, Gauhar AR, Nakhaei-Rad S, Kordes C, Koessmeier KT, Cirstea IC, Olayioye MA (2014). Functional cross-talk between ras and rho pathways: a Ras-specific GTPase-activating protein (p120RasGAP) competitively inhibits the RhoGAP activity of deleted in liver cancer (DLC) tumor suppressor by masking the catalytic arginine finger. J. Biol. Chem.

[49] Hancock JF, Cadwallader K, Paterson H, Marshall CJ (1991). A CAAX or a CAAL motif and a second signal are sufficient for plasma membrane targeting of ras proteins. EMBO J.

[50] Choy E, Chiu VK, Silletti J, Feoktistov M, Morimoto T, Michaelson D, Ivanov IE, Philips MR (1999). Endomembrane trafficking of ras: the CAAX motif targets proteins to the ER and Golgi. Cell.

[51] Moffat J, Grueneberg DA, Yang X, Kim SY, Kloepfer AM, Hinkle G, Piqani B, Eisenhaure TM, Luo B, Grenier JK, Carpenter AE, Foo SY, Stewart SA (2006). A lentiviral RNAi library for human and mouse genes applied to an arrayed viral high-content screen. Cell.

