# Supplementary material for: The anticancer phytochemical rocaglamide inhibits Rho GTPase activity and cancer cell migration
Source: Oncotarget. 2016 Jun 20;7(32):51908–21. doi: 10.18632/oncotarget.10188 (PMC5239523; doi:10.18632/oncotarget.10188)
Supplement: Supplementary file 1 [file oncotarget-07-51908-s001.pdf]

The anticancer phytochemical rocaglamide inhibits Rho GTPase activity and cancer cell migration

SUPPLEMENTARY MATERIALS

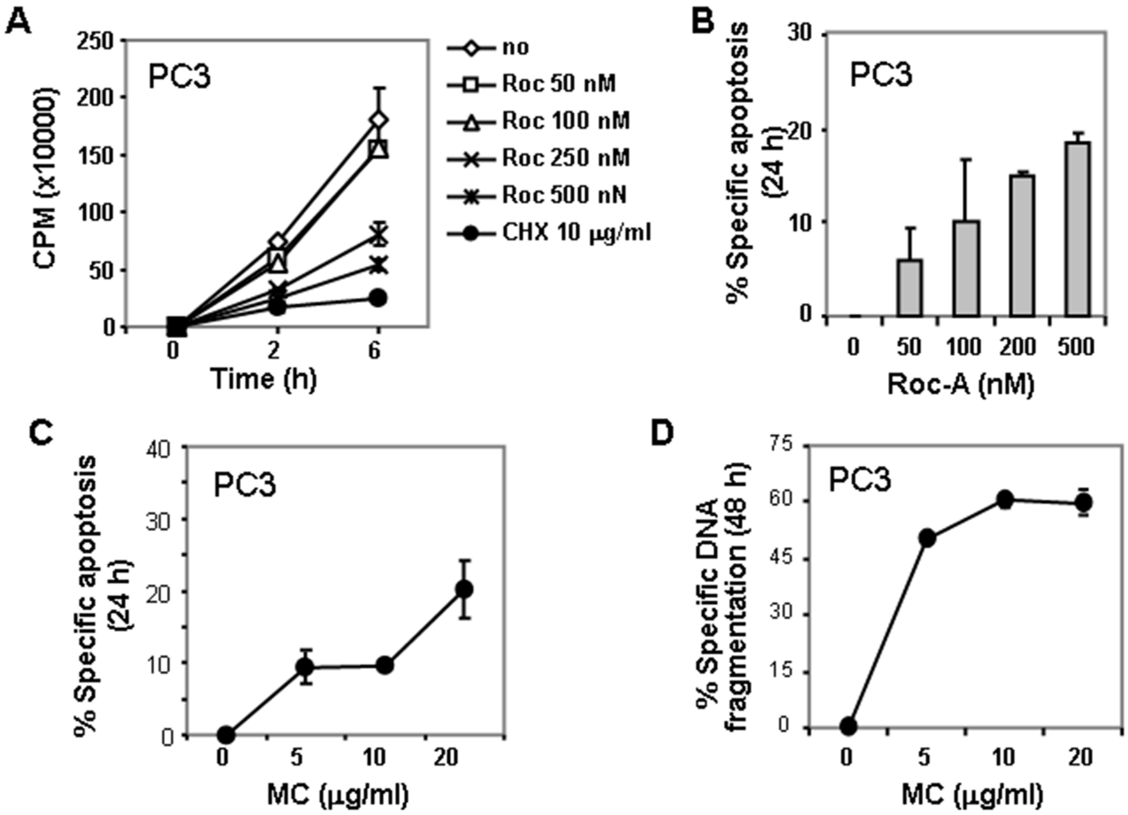

Supplementary Figure S1: Analysis of the effects of Roc-A and Mitomycin C (MC) on translation inhibition and apoptosis induction in PC3 cells. A. Effects of Roc-A and MC on translation inhibition in PC3 cells. **B**. Effects of Roc-A on apoptosis induction in PC3 cells. **C.** Effects of MC on apoptosis induction in PC3 cells at 24 h (AnxV/7aad staining). **D.** Effects of MC on apoptosis induction in PC3 cells at 48 h. (DNA fragmentation).

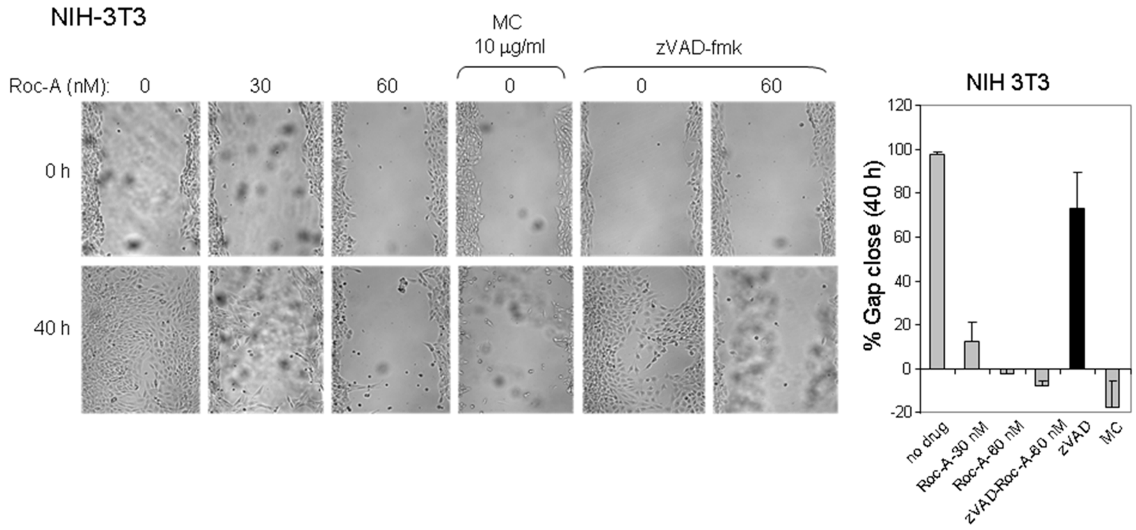

Supplementary Figure S2: Roc-A inhibits migration of the mouse non-tumor fibroblast cell line NIH-3T3. The experiment was carried out as described in Figure 1 and 2.

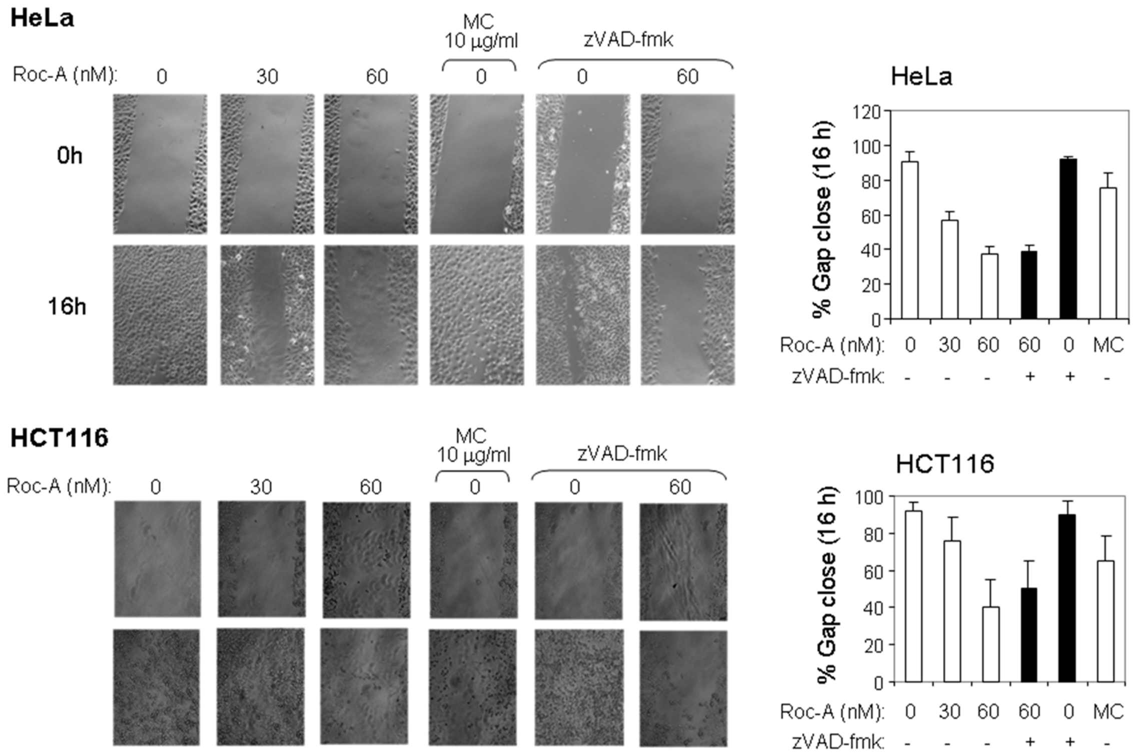

Supplementary Figure S3: Roc-A inhibits migration of the human cervical HeLa cancer cells and the human colon HCT116 cancer cells. The experiments were carried out as described in Figure 1 and 2.

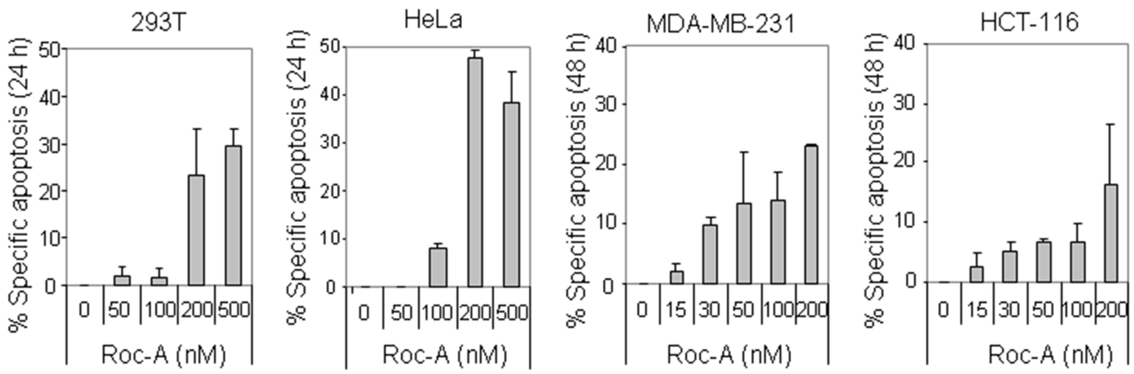

Supplementary Figure S4: Effects of Roc-A on cell viability in different cancer cell lines. Cancer cell lines used in the wound-assay analysis were treated with different doses of Roc-A (A) for the indicated times. Apoptotic cell death was examined by AnxV and 7aad staining.

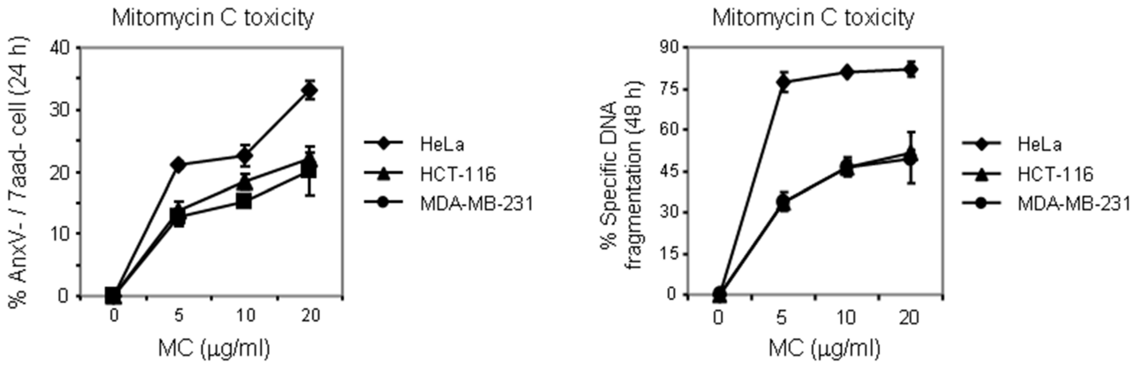

Supplementary Figure S5: Effects of Mitomycin C (MC) on cell viability in different cancer cell lines. Cancer cell lines used in the wound-assay analysis were treated with different doses of MC for 24 or 48 h as indicated. MC-induced apoptotic cell death was determined by either AnxV / 7aad staining (at 24 h) or DNA fragmentation (at 48 h).

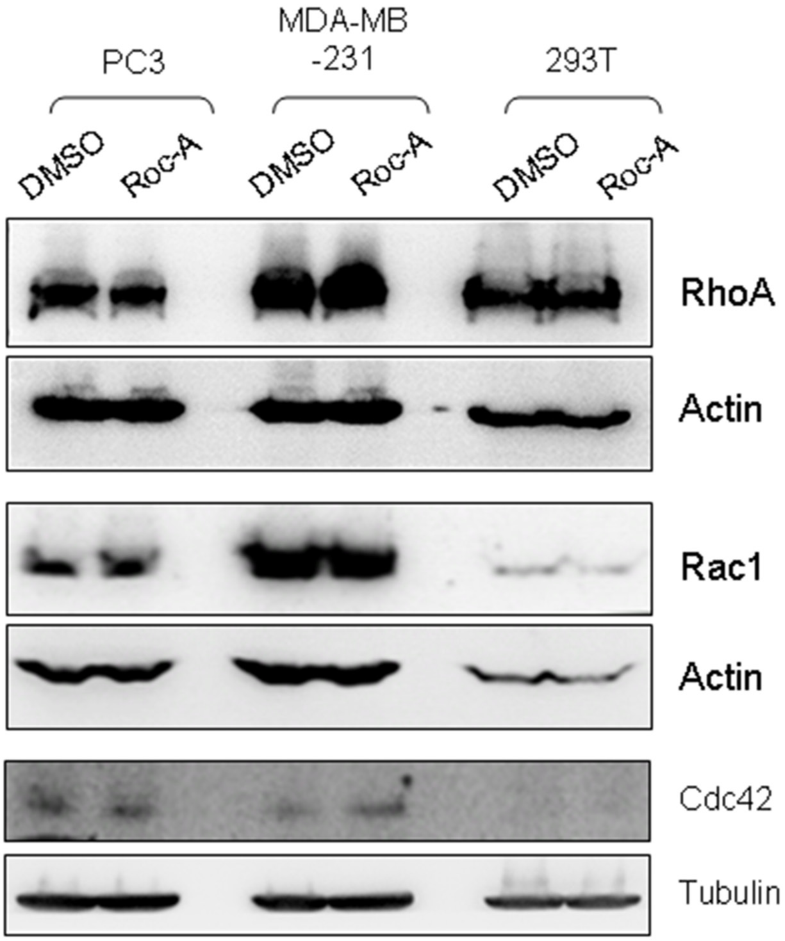

Supplementary Figure S6: The protein expression levels of Rho GTPases are not affected by Roc-A. PC3, MDA-MB-231 and 293T cells were treated with 50 nM of Roc-A for 24 h. Cell lysates were subjected to immunoblot analysis for the expression levels of RhoA, Rac1 and Cdc42. Data represent one of two independent experiments.

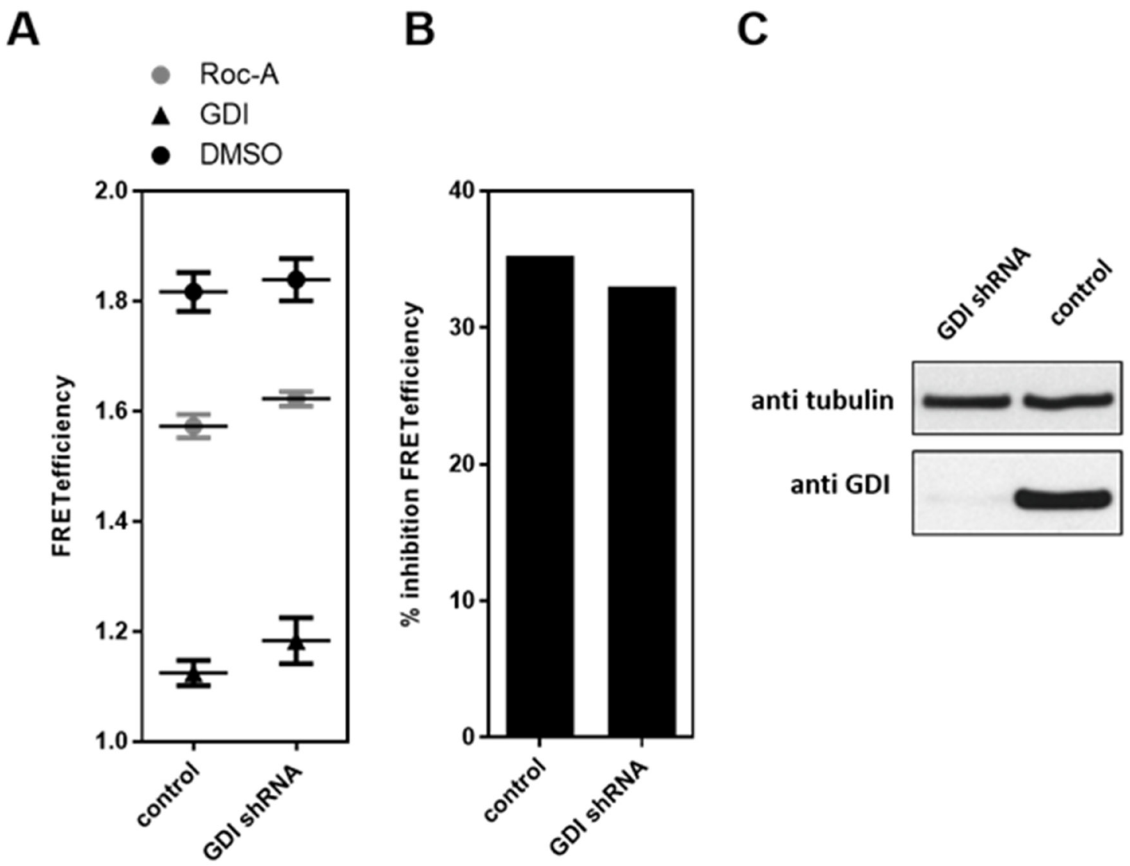

Supplementary Figure S7: Effect of GDI knockdown on Roc-A-mediated inhibition of RhoA activity. A. GDI was knocked down in 293T cells, followed by overexpression of RhoA FRET sensors either with a control plasmid (Control, 30 nM Roc-A) or together with GDI. Cells were treated with 30 nM Roc-A or vehicle (DMSO for Control and GDI) for 24h. Results are an average of three independent experiments. Error bars (S.E.M.) are shown. **B.** FRET efficiency from B was normalized to GDI and DMSO values and % inhibition FRET efficiency was calculated. **C.** Western blot showing the expression levels of GDI in GDI shRNA- and control-transfected cells.

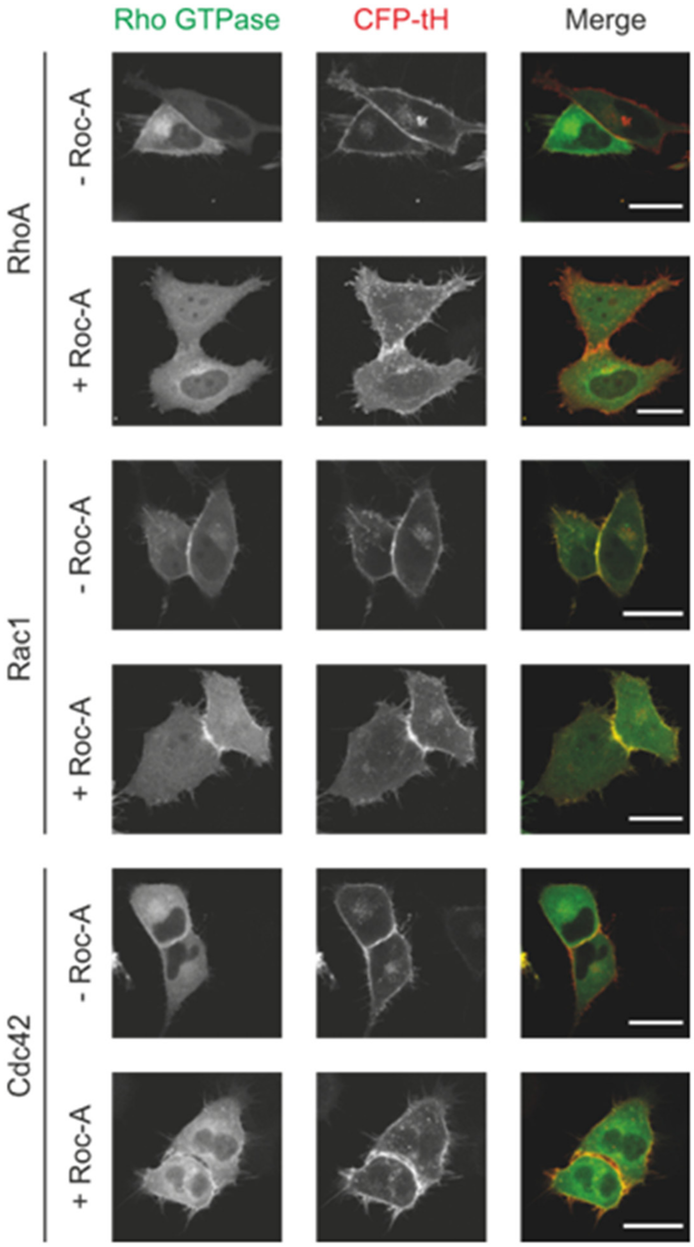

Supplementary Figure S8: Roc-A has no effect on cellular localization of Rho GTPases RhoA, Rac1 and Cdc42 in HeLa cells. mCitrine-RhoA, -Rac1 or -Cdc42 (green) were overexpressed in HeLa cells each together with the cell membrane marker mCerulean-tH (red). Cells were treated with 30 nM Roc-A or vehicle (DMSO) for 24h. Representative images (Z-slices) are shown. Scale bar = 20 µm.

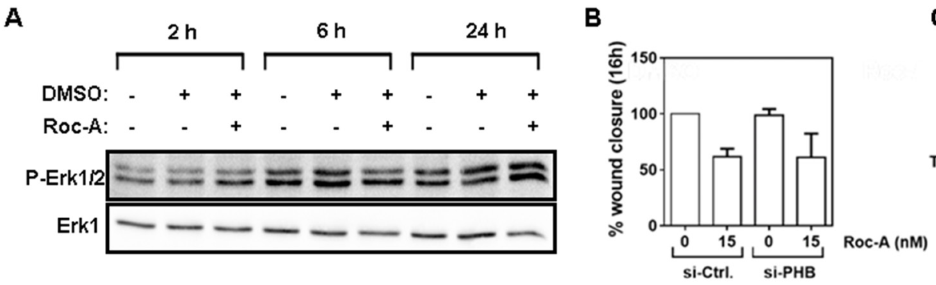

Supplementary Figure S9: Roc-A does not inhibit cell migration *via* PHB. A. Roc-A does not influence Erk phosphorylation at low concentrations. PC-3 cells were treated with solvent (DMSO) or Roc-A (15 nM) for the indicated time periods. The phosphorylation of Erk and total Erk expression levels were analyzed by Western blotting. Results are representative of two independent experiments. **B.** The level of PHB expression does not influence the anti-migratory effect of Roc-A. PC-3 cells were transiently transfected with siRNA against PHB and 24h later used in wound-assays. Control-transfected cells and PHB-si-RNA-transfected cells were treated with solvent (DMSO) or Roc-A as indicated and the percentage of gap closure was quantified 16h following treatment. Results are an average of three independent experiments. Error bars (S.D.) are shown. **C.** Western blot analysis of the efficiency of PHB knockdown. A representative blot is shown. Protein lysates were analyzed 48h post-transfection. Results correspond to B and are representative of three independent experiments.
